# Diurnal and Seasonal Variations in Carbon Dioxide Exchange in Ecosystems in the Zhangye Oasis Area, Northwest China

**DOI:** 10.1371/journal.pone.0120660

**Published:** 2015-03-24

**Authors:** Lei Zhang, Rui Sun, Ziwei Xu, Chen Qiao, Guoqing Jiang

**Affiliations:** 1 State Key Laboratory of Remote Sensing Science, Jointly Sponsored by Beijing Normal University and the Institute of Remote Sensing Applications, CAS, Beijing, China; 2 School of geography and Remote Sensing Sciences, Beijing Normal University, Beijing, China; 3 Beijing Key Lab for Remote Sensing of Environment and Digital Cities, Beijing, China; University of Illinois at Urbana-Champaign, UNITED STATES

## Abstract

Quantifying carbon dioxide exchange and understanding the response of key environmental factors in various ecosystems are critical to understanding regional carbon budgets and ecosystem behaviors. For this study, CO_2_ fluxes were measured in a variety of ecosystems with an eddy covariance observation matrix between June 2012 and September 2012 in the Zhangye oasis area of Northwest China. The results show distinct diurnal variations in the CO_2_ fluxes in vegetable field, orchard, wetland, and maize cropland. Diurnal variations of CO_2_ fluxes were not obvious, and their values approached zero in the sandy desert, desert steppe, and Gobi ecosystems. Additionally, daily variations in the Gross Primary Production (*GPP*), Ecosystem Respiration (*R_eco_*) and Net Ecosystem Exchange (*NEE*) were not obvious in the sandy desert, desert steppe, and Gobi ecosystems. In contrast, the distributions of the *GPP*, *R_eco_*, and *NEE* show significant daily variations, that are closely related to the development of vegetation in the maize, wetland, orchard, and vegetable field ecosystems. All of the ecosystems are characterized by their carbon absorption during the observation period. The ability to absorb CO_2_ differed significantly among the tested ecosystems. We also used the Michaelis-Menten equation and exponential curve fitting methods to analyze the impact of Photosynthetically Active Radiation (*PAR*) on the daytime CO_2_ flux and impact of air temperature on *R_eco_* at night. The results show that *PAR* is the dominant factor in controlling photosynthesis with limited solar radiation, and daytime CO_2_ assimilation increases rapidly with *PAR*. Additionally, the carbon assimilation rate was found to increase slowly with high solar radiation. The light response parameters changed with each growth stage for all of the vegetation types, and higher light response values were observed during months or stages when the plants grew quickly. Light saturation points are different for different species. Nighttime *R_eco_* increases exponentially with air temperature. High Q_10_ values were observed when the vegetation coverage was relatively low, and low Q_10_ values occurred when the vegetables grew vigorously.

## Introduction

Net Ecosystem Exchange (*NEE*) is the net change in carbon stored in an ecosystem caused by photosynthesis, carbon storage in the canopy air as well as biological and non-biological respirations that lead to carbon emissions [1].The interplay between these processes leads to carbon balance in an ecosystem. Every year, approximately 14% of the carbon in the atmosphere is exchanged with the terrestrial biosphere [2, 3]. Knowledge of the regional carbon budget and how it changes over time is critical for understanding the mechanisms that control the global terrestrial carbon cycle and the sustainability of current carbon sinks. It is evident that different terrestrial surfaces contribute to regional carbon budgets differently due to the heterogeneity of the ecosystems [4]. Arid and semi-arid landscapes cover more than one-third of China [5]. However, the land surface-atmosphere carbon flux processes in arid areas have received much less attention compared to other regions [6–8]. Currently, the most common method for measuring carbon flux is the eddy covariance (EC) technique, which provides a reliable and non-destructive approach to measure the *NEE* directly in an ecosystem [6]. This method has been widely used in various terrestrial ecosystems, including temperate coniferous and boreal forests, crops, grasslands, chaparrals, wetlands, and tundras [9, 10].

The Heihe River basin is located in northwest China and is an arid area that is sensitive to climate changes. The region is important with respect to land-atmosphere interactions because of its unique energy budget and quickening aridification and desertification [11, 12]. A study and comparison of the CO_2_ flux and environmental responses in various ecosystems in this area will help us better understand carbon cycle in arid regions and the impact of human activity on it.

**Fig 1:**
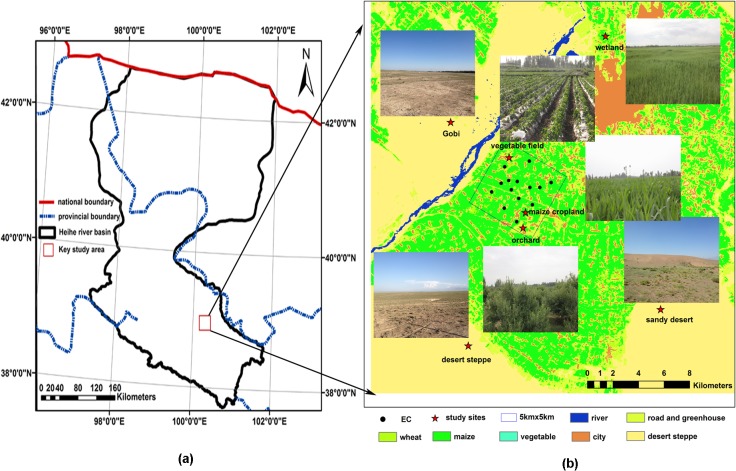
The distribution of the eddy covariance in different ecosystems.

To recognize the processes and mechanisms of the eco-hydrological system in Heihe River basin, the Heihe Plan has been carried out since 2010 to integrate observation, data management, and model simulation of both the physical and socioeconomic processes [13]. Heihe Watershed Allied Telemetry Experimental Research (HiWATER) program was designed as a comprehensive eco-hydrological experiment within the framework of the Heihe Plan; it is based on the diverse needs of interdisciplinary studies within the plan and the existing observational infrastructures in the Heihe River basin [13]. The MultiScale Observation Experiment on Evapotranspiration over heterogeneous land surfaces is a thematic experiment in HiWATER (HiWATER-MUSOEXE), which was conducted from June 2012 to September 2012 in the middle reach of the Heihe River basin, and involved a flux observation matrix. HiWATER-MUSOEXE is composed of two nested matrices: one large experimental area (30 km × 30 km) and one kernel experimental area (5.5 km × 5.5 km). The large experimental area contained one superstation in the oasis cropland and four ordinary stations around the oasis with sandy desert, desert steppe, Gobi, and wetland surfaces. The 5.5 km × 5.5 km kernel experimental area was located in the oasis and contained one vegetable field, one orchard, one village and 14 maize ordinary stations (Fig. 1B). The difference between a superstation and ordinary station is the meteorological profile (i.e., 7 layer wind speed/direction and air temperature/humidity) and more observations (i.e., 2 layers of EC) were taken at the superstation. Overall, 22 EC system sets and 21 automatic weather station sets were involved in the experiment (there were 2 ECs installed at the superstation at different heights) (Fig. 1B).

The objectives of the study were to (1) compare the characteristics of diurnal and daily variations in the CO_2_ flux across different ecosystems; (2) quantify the CO_2_ flux using metrics, including the gross primary production (*GPP*), ecosystem respiration (*R*_*eco*_), and *NEE* metrics; and (3) analyze the primary factors that control diurnal and daily variations in CO_2_ exchanges in the middle reach of the Heihe River basin.

## Methods

### Ethics statement

Our experiments were associated with the HiWATER-MUSOEXE. We used seven sites to conduct our study, which encompassed a vegetable field, orchard, wetland, Gobi, sandy desert, desert steppe and a maize cropland. No specific permissions were required to our sites. The studied areas did not involve endangered or protected species. The specific locations of our study are as follows: Vegetable field (100.35816 E, 38.89322 N), Orchard (100.36974 E, 38.84512 N), Maize cropland (100.37225 E, 38.85557 N), Gobi (100.30420 E, 38.91496 N), Sandy desert (100.49330 E, 38.78917 N), Desert steppe (100.31860 E, 38.76519 N), Wetland (100.44640 E, 38.97514 N).

### Sites description

The study area, Zhangye oasis irrigation area, is located in Gansu province, northwest China, which is in the middle of the Hexi Corridor (Fig. 1A). The Heihe River runs through the entire territory, forming a unique desert oasis (Fig. 1A). The annual mean air temperature is approximately 6°C, and the warmest month is July. The annual mean precipitation is approximately 114.9 mm; more than 70% of this precipitation is accumulated during the growing season from June to September. The annual frost-free period lasts approximately 169 days [14].

The Zhangye oasis irrigation area is surrounded by sandy desert, wetland, Gobi and desert steppe ecosystems and is a typical irrigated agricultural area [13]. Fig. 1A shows the location of the focus area for this study. Seven sites, with orchard, vegetable field, wetland, desert steppe, Gobi, sandy desert, and maize surfaces, were included in this study. Fig. 1B shows the surfaces at different sites. Each site is fairly open and flat and the fetch is approximately 100 m- 200 m in all directions. The prevailing wind direction is northwesterly. The area of the orchard (EC location) is approximately 53 hm^2^, and consists mainly of planted apple trees. Chilies are the main vegetable in the vegetable field, which has an area of 21.6 hm^2^. *Phragmites australis* are the dominant species in the wetland, which has a total area of nearly 1733 hm^2^. The dominant vegetation in the desert steppe is *Salsola passerina*, while in the Gobi, the vegetation is sparse and dominated by *Compositae* and *Salsola passerina*. In contrast, there is slightly more vegetation in the sandy desert ecosystem, which is dominated by drought-tolerant vegetation, such as *Peganum harmala* and *Tribulus terrestris*. Maize is the main crop in the irrigation area. The superstation is located in a maize cropland field with an area of 200 hm^2^. During the studied timeframe, irrigation occurred once a month in the orchard, vegetable field and maize cropland.

### Measurements and data acquisition

The fluxes of CO_2_ were continuously measured using EC systems. The data used in the study spanned from June 10 in 2012 to September 14 in 2012, except for in the wetland where the data spanned from June 26 in 2012 to September 14 in 2012. The EC system used consists of a three-dimensional sonic anemometer (CSAT-3/Gill, Campbell Scientific Instruments Inc., USA/Gill, UK) and an open-path infrared gas analyzer (LI-7500A, Licor Inc., USA), which measure the three components of wind speed, sonic virtual temperature, and the concentrations of water vapor and CO_2_. The data were recorded with a data logger (CR1000/CR3000/CR5000, Campbell Scientific Instruments Inc.) and CF card at a frequency of 10 Hz. Air temperature and relative humidity (HMP45C, Vaisala Inc., Helsinki, Finland) were measured 5 and 10 m above the ground. Photosynthetically Active Radiation (*PAR*) (LI-190SA, LI-COR Inc.) was measured above the canopy at a height of 12 m. Rainfall was measured using a tipping bucket rain gauge (TE525MM, Campbell Scientific Instruments Inc.). The soil temperature (Campbell-107, Campbell Scientific Instruments Inc.) and volumetric soil water content (TRIMEEZ/IT, IMKO, Ettlingen, Germany) were measured at 0.02, 0.04, 0.10, 0.20, 0.40, 0.60 and 1.0 m depths in all plots. In the analysis, we use the soil temperature and water content measurements from a depth of 0.04 m and the air temperature and relative humidity from a height of 5 m. Because of the different vegetation conditions and instrument types, the heights of the EC systems are not the same among the different ecosystems. The heights for the EC systems were 7 m, 2.85 m, 3.8 m, 4.6 m, 4.6 m, 5.2 m, and 4.5 m in the orchard, desert steppe, vegetable field, Gobi, sandy desert, wetland, and maize cropland ecosystems, respectively.

### Data processing and gap filling

Post-processing calculations were performed using the Edire software package (http://www.geos.ed.ac.uk/abs/research/micromet/EdiRe) and included outlier exclusion, time delay corrections, density fluctuations, spectral loss, secondary coordinate rotation and sonic virtual temperature conversion [15–18].

Due to the atmospheric stability of the weather and the physical constraints of the impact apparatus, the data retained implausible points after this processing procedure. Therefore, we used the following procedure to further process the data.

(1) Removed the data from one hour before and after rainy periods [19]. (2) Excluded the negative data collected during the night [20, 21]. (3) Removed the data that were outside the instruments’ measurement range or outside the reasonable range of values [20, 21]. The range for the different ecosystems was not the same. (4) Removed the data for which the friction velocity (u) was less than the threshold friction velocity in night [20, 21]. The threshold friction velocity is different between the ecosystems, with 0.15 m·s^−1^ for the orchard, 0.14 m·s^−1^ for the maize cropland, 0.13 m·s^−1^ for the wetland and sandy desert, 0.10 m·s^−1^ for the vegetable field, Gobi and steppe desert.

*NEE* is calculated from the sum of the EC flux (*F*_*c*_) and the storage term (*F*_*s*_). *F*_*s*_ is the flux associated with the CO_2_ stored in the layer below the level in which CO_2_ flux measurements are taken. The value of *F*_*s*_ is obtained by integrating the change in CO_2_ concentration in the air layer up to the height of the EC [22]. Because the flux measurement systems were not high at the studied sites, the storage term *F*_*s*_ was usually smaller than *F*_*c*_ and the daily values tended to be zero, so *F*_*s*_ is neglected in the calculation of *NEE*. *GPP* is the difference between the estimated ecosystem respiration (*R*_*eco*_) and observed *NEE*:

$$GPP\;=\;R_{\text{eco}}-NEE$$ (1)

*GPP* (mg CO_2_·m^−2^·s^−1^) represents CO_2_ assimilated by photosynthesis and *R*_*eco*_ (mg CO_2_·m^−2^·s^−1^) consists of respiratory CO_2_ released from the soil and vegetation.

Nighttime *NEE* values are equal to *R*_*eco*_ because the *GPP* equals zero at night. Daytime estimates of *R*_*eco*_ were obtained from the nighttime *NEE*-temperature relationship. The relationship between nighttime *NEE* and air temperature can be described by the Vant Hoff equation (Eq. 2) [23],

$$R_{\text{eco}}\;=\;ae\;{}^{bT}$$ (2)

where *a* and *b* are regression parameters and *T* is the air temperature (°C). The temperature sensitivity coefficient (Q_10_) was determined from the following equation:

$$Q_{\;10}\;=\;e^{\;10b}$$ (3)

Additionally, missing nighttime data were also estimated by an exponential regression, using Eq. 2, of the measured nighttime *R*_*eco*_ and air temperature. In this study, we used the air temperature as the fitting parameter because it yielded better results (higher R^2^ values) than the soil temperature.

There are five basic models used for describing the partial dependence of the *NEE* on the *PAR* [24]. The Michaelis–Menten rectangular hyperbola model (Eq. 4) [25, 26] is typically used.

$$NEE\;=\;R_{e\text{co}}\;-\;\frac{P_{max}PAR}{K_{\;m}\;+\;PAR}$$ (4)

where *PAR* (μmol· m^−2^·s^−1^) is the incident photosynthetically active radiation, *P*_*max*_ (mg CO_2_· m^−2^·s^−1^) is the maximum CO_2_ flux (with infinite light) and *K*_*m*_ is the level of *PAR* at which *NEE* is one half of *P*_*max*_. *R*_*eco*_ (mg CO_2_· m^−2^·s^−1^) is the average daytime respiration. The apparent quantum yield (α) can be calculated as follows [27]:

$$\alpha \;=\;\frac{P_{\;max}}{K_{\;m}}$$ (5)

The missing or bad data for the daytime were gap-filled using the ‘look-up’ table method, which is based on the empirical relationships created for each site between *NEE*, air temperature and incident PAR when meteorological data are available [19]. Previous studies have shown that the daily change of CO_2_ flux is associated to the temperature, soil moisture and vegetation phenology [28]. To make the gap-fill more rational, we interpolated the missing data by the month in the orchard, wetland, and vegetable field ecosystems and by the growth stage in the maize cropland. Four different growth stages were defined according to the maize phenology: seeding stage (June 10–19), jointing stage (June 20-July 20), filling stage (July 21-August 5) and mature stage (August 6-September 15). Considering that seven leaves were growing at the beginning of our observation, it was understood that the seeding stage in our study did not include the prior period.

## Results and Discussion

### Diurnal variations in *NEE*

**Fig 2:**
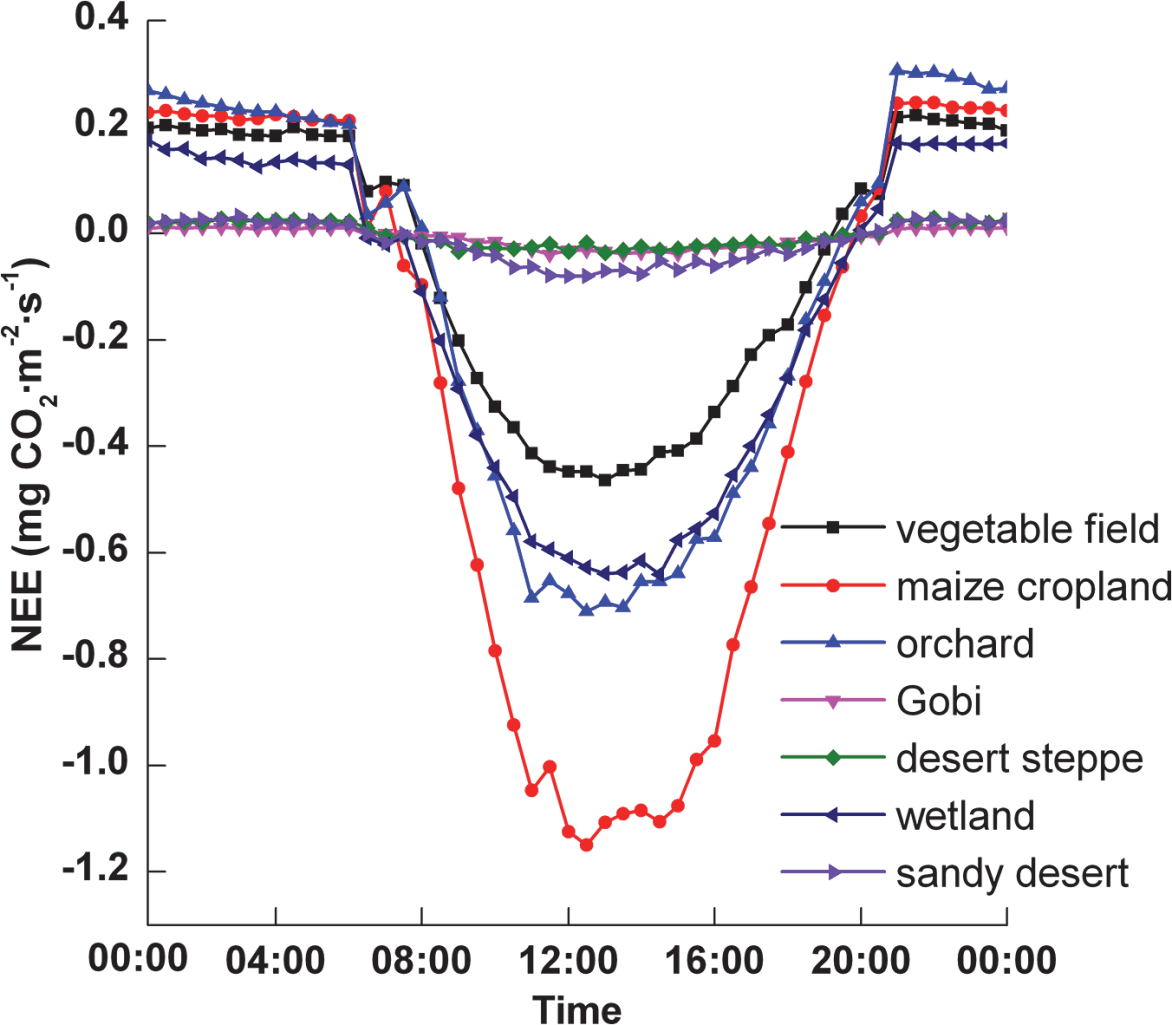
Diurnal variations of *NEE* in different ecosystems.

Based on the observations from the study period (i.e., June 10—September 14, and, in wetland, June 26—September 14), the diurnal variations of *NEE* were calculated for different ecosystems. Fig. 2 shows the diurnal variations of *NEE* for different ecosystems, which were calculated using the average flux during the observation period (positive values represent CO_2_ emissions; negative values represent CO_2_ absorption). As seen in Fig. 2, the diurnal variations in *NEE* from the different ecosystems show a single peak. The values of *NEE* are negative in the daytime and positive at nighttime, and the minimum value occurs around noon. The negative daytime values of *NEE* indicate that CO_2_ assimilation via photosynthesis is greater than CO_2_ release from the respiratory processes of ecosystems. During the nighttime, however, CO_2_ is released to the atmosphere via respiratory processes in the soil and plants, and the *NEE* values are then positive.

However, there were differences among the ecosystems. CO_2_ absorption and emission in sandy desert, desert steppe, and Gobi ecosystems were very weak, and the values were close to zero, meaning the diurnal variations of *NEE* were not significant. Conversely, the diurnal variations of *NEE* were significant in the vegetable field, orchard, maize cropland, and wetland ecosystems. There was an extremely small CO_2_ flux in the sandy desert, desert steppe and Gobi ecosystems; the maximum net CO_2_ absorptions were 0.081, 0.036, 0.038 mg CO_2_·m^−2^·s^−1^, respectively. Carbon absorption during the daytime was similar in desert steppe and Gobi, but the nighttime carbon emission was higher in the desert steppe than in the Gobi. This is because the Gobi surface contains more gravel than the desert steppe, resulting in relatively low soil respiration at night. There was a relatively large fluctuation in the maize cropland, orchard, wetland and vegetable field ecosystems; the minimum values were −1.15, −0.71, −0.64 and −0.46 mg CO_2_·m^−2^·s^−1^, respectively. The peak CO_2_ uptake for the maize cropland was approximately 1.6 times the value observed in the orchard, 1.8 times the wetland value and 2.5 times the vegetable field value.

### Daily variation in *GPP*, *R*_*eco*_ and *NEE*

**Fig 3:**
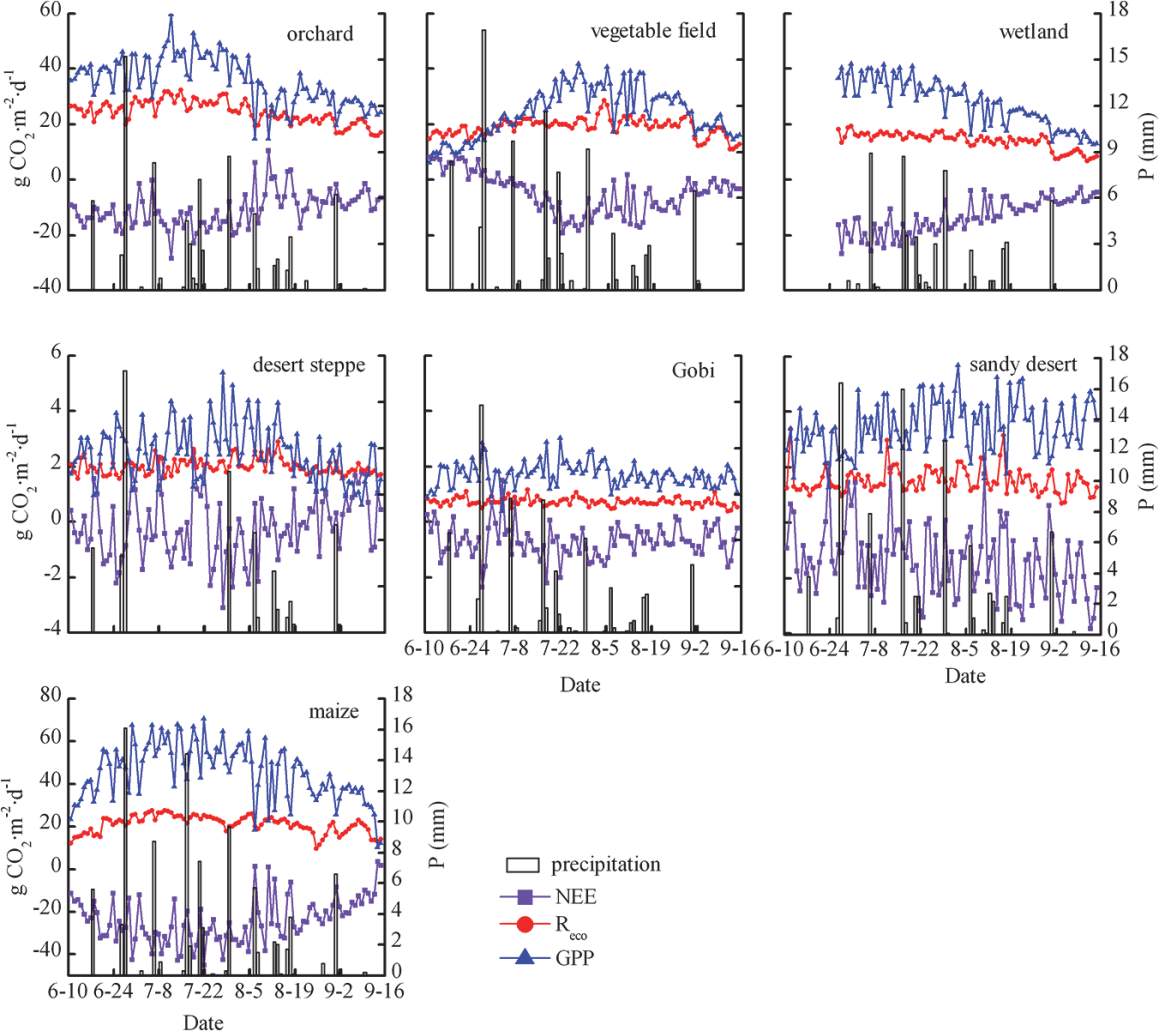
Daily variations in *NEE*, *R*_*eco*_ and *GPP* in different ecosystems.

Values of *NEE*, *R*_*eco*_, and *GPP* for different ecosystems over the observation period are shown in Fig. 3. Because sparse and drought-tolerant vegetation dominate the sandy desert, desert steppe and Gobi ecosystems, the values of *NEE*, *GPP*, and *R*_*eco*_ were much lower in these ecosystems than in other ecosystems. Meanwhile, daily *NEE*, *GPP*, and *R*_*eco*_ variations were not evident. However, there were significant daily variations in *NEE*, *GPP*, and *R*_*eco*_ in the vegetable field, orchard, maize cropland, and wetland ecosystems, which are closely related to the growth and phenology of the vegetation.

The minimum daily *NEE* values were −3.11, −2.40, and −3.82 g CO_2_·m^−2^·d^−1^ in the desert steppe, Gobi and sandy desert ecosystems, respectively, and occurred in July in these three ecosystems (Table 1). The maximum values of *R*_*eco*_ were 2.90, 1.14, and 3.37 g CO_2_·m^−2^·d^−1^, while the maximum values of *GPP* were 5.41, 3.41, and 5.68 g CO_2_·m^−2^·d^−1^ in the desert steppe, Gobi and sandy desert ecosystems, respectively (Table 1). The total *NEE*, *R*_*eco*_ and *GPP* during the observation period in the desert steppe, Gobi and sandy desert ecosystems are shown in Table 2. The total *NEE* were −67.9, −129.6 and −17.8 g CO_2_·m^2^ in the Gobi, sandy desert and desert steppe, respectively. Thus, these three ecosystems acted as carbon sinks during the observation period. The *GPP/R*_*eco*_ ratio in these three ecosystems is greater than 1 (Table 2), which also illustrates their carbon sink nature. These results are consistent with previous studies because many studies also found that the desert ecosystem is a carbon sink and the value of total *NEE* in our study is within the observed range from previous studies. For example, Liu et al. [29] found that the saline desert ecosystem in western China was a significant carbon sink and the values of *NEE* were −236.2 g CO_2_·m^−2^, −63.1 g CO_2_·m^−2^, and −92.0 g CO_2_·m^−2^ during the growing seasons of 2004, 2005, and 2006 respectively. Wohlfahrt et al. [30] measured the Mojave Desert ecosystem in the USA and found that it was a CO_2_ sink, taking up 102±67 and 110±70 g C·m^−2^ in 2005 and 2006, respectively.

**Table 1:** Maximum *GPP*, *R*_*eco*_, and Minimum *NEE* for different ecosystems during the observation period (June 10—September 14, and, in wetland, June 26—September 14).

Sites	Vegetable field	Orchard	Maize	Wetland	Gobi	Sandy desert	Desert steppe
Minimum NEE (g CO_2_·m^−2^·d^−1^)	−19.2	−28.4	−45.3	−26.8	−2.4	−3.82	−3.11
Maximum R_eco_ (g CO_2_·m^−2^·d^−1^)	28.7	32.3	27.5	19.4	1.14	3.37	2.9
Maximum GPP (g CO_2_·m^−2^·d^−1^)	41.8	59.8	70.8	41.7	3.41	5.68	5.41

**Table 2:** Total *GPP*, *R*_*eco*_, and *NEE* for different ecosystems during the observation period (June 10—September 14, and, in wetland, June 26—September 14)

sites	Month/Stage	Fitting equation	R^2^	Q_10_
Vegetable field	June	*R_eco_* = 0.0619exp(0.047*T)	0.119	2.05
	July	*R_eco_* = 0.113exp(0.0336*T)	0.083	1.40
	August	*R_eco_* = 0.0733exp(0.0548*T)	0.206	1.73
	September	*R_eco_* = 0.0697exp(0.0498*T)	0.265	1.65
Orchard	June	*R_eco_* = 0.0841exp(0.0571*T)	0.235	1.77
	July	*R_eco_* = 0.0987exp(0.0552*T)	0.210	1.74
	August	*R_eco_* = 0.1014exp(0.0451*T)	0.234	1.57
	September	*R_eco_* = 0.1287exp(0.0323*T)	0.375	1.38
Wetland	July	*R_eco_* = 0.0741exp(0.0402*T)	0.132	1.49
	August	*R_eco_* = 0.0605exp(0.0437*T)	0.175	1.55
	September	*R_eco_* = 0.048exp(0.0404*T)	0.305	1.50
Desert steppe	June-September	*R_eco_* = 0.0028exp(0.0235*T)	0.009	1.26
Gobi	June-September	*R_eco_* = 0.0026exp(0.043*T)	0.021	1.54
Sandy Desert	June-September	*R_eco_* = 0.0036exp(0.00572*T)	0.021	1.77
Maize cropland	seedling stage	*R_eco_* = 0.0316exp(0.0718*T)	0.312	2.05
	jointing stage	*R_eco_* = 0.1229exp(0.0392*T)	0.126	1.48
	filling stage	*R_eco_* = 0.1055exp(0.056*T)	0.512	1.75
	mature stage	*R_eco_* = 0.1052exp(0.0407*T)	0.293	1.50

In comparison, the fluctuations of *NEE* in the maize cropland, orchard, wetland, and vegetable field ecosystems were large during the observation period (Fig. 3). On rainy days, the ecosystems become sources of CO_2_ because the *NEE* is primarily composed of soil and plant respiration (Fig. 3). The *NEE* values were negative throughout the observation period in the orchard, maize cropland and wetland ecosystems. However, at the beginning of the observation, *NEE* was positive in vegetable field, but gradually decreased and became slightly negative by the end of June. This is likely explained by the fact that the chilies in the vegetable field had been recently transplanted and grew very slowly at the beginning of our observation. The minimum daily *NEE* was observed in July, at which point the values were −45.3, −28.4, −26.8 and −19.2 g CO_2_·m^−2^·d^−1^ in the maize cropland, orchard, wetland and vegetable field ecosystems, respectively (Table 1). The maximum *GPP* values were 70.8, 59.8, 41.7, 41.8 g CO_2_·m^−2^·d^−1^ and the maximum *R*_*eco*_ values were 27.5, 32.3, 19.4, 28.7 g CO_2_·m^−2^·d^−1^ in the maize cropland, orchard, wetland and vegetable field ecosystems, respectively (Table 1). The total *NEE*, *R*_*eco*_ and *GPP* for the maize cropland, orchard, wetland, and vegetable field ecosystems throughout the entire observation period are shown in Table 2. During the observation period, the *GPP/R*_*eco*_ ratios were higher than 1 (Table 2), which implies that *NEE* is dominated by *GPP* and that *PAR* is a dominant factor controlling *NEE*. During the observation period, the total *NEE* for the maize cropland, orchard, wetland and vegetable field were −2342.6, −1050.3, −1085.1, and −468.1 g CO_2_·m^2^, respectively (Table 2). Obviously, these ecosystems are characterized by CO_2_ sinks. The carbon absorption capacities of various ecosystems are significantly different. The integrated order of the carbon absorption capacity in each ecosystem is: maize cropland > wetland > orchard > vegetable field> sandy desert> Gobi> desert steppe.

The maximum net CO_2_ uptake reported from our site was compared with other ecosystems. There are major differences regarding the daily maximum net CO_2_ uptake among different crops. In our study, CO_2_ uptake in the maize cropland was slight greater than 38.47 g CO_2_·m^−2^·d^−1^, the value measured in an oasis field maize ecosystem in Xinjiang by Zhao [31], and was similar to the uptake in a maize ecosystem in the North China Plain (45.8 g CO_2_·m^−2^·d^−1^) [32]. However, the value observed in this study is lower than that obtained by Baker and Griffis [33] in a maize/soybean ecosystem in the USA (51.3 g CO_2_·m^−2^·d^−1^) and is also lower than the value (67.8 g CO_2_·m^−2^·d^−1^) measured by Hollinger in a maize and soybean rotation agriculture ecosystem in the USA [34]. Precipitation may be one of the reasons for the carbon uptake difference. The annual mean precipitation of the two sites in the USA is more than 570 mm, which is significantly higher than in our studied area. Meanwhile, different maize species and tillage practices may also result in differences in the CO_2_ flux.

There is less research being conducted regarding CO_2_ flux in vegetable fields. The net CO_2_ uptake in the vegetable field is higher than in wheat (15.62 g CO_2_·m^−2^·d^−1^) and cotton ecosystems (8.96 g CO_2_·m^−2^·d^−1^) in Xinjiang according to Zhao [31], but lower than in the wheat ecosystem (30.0 g CO_2_·m^−2^·d^−1^) in the North China Plain [32]. The daily maximum net CO_2_ uptake in the apple orchard in our study is similar to that from a vineyard ecosystem (28.9 g CO_2_·m^−2^·d^−1^) in northwestern China [35]. However, the value is higher than in a broad-leaved Korean pine forest (22.3 g CO_2_·m^−2^·d^−1^) and a Dahurian larch forest (15.8 g CO_2_·m^−2^·d^−1^) in northeast China [36]. This is because the environmental conditions in artificial ecosystems are better than those in a natural ecosystem.

The daily maximum net CO_2_ absorption in the wetland was higher than 14.3 g CO_2_·m^−2^·d^−1^ measured in an alpine wetland ecosystem on the Qinghai-Tibetan Plateau [37]. The value is within the observed range from wetlands on Chongming Island of Shanghai (18.3 g CO_2_·m^−2^·d^−1^–44.0 g CO_2_·m^−2^·d^−1^) [38]. During the observation period, the wetland was a strong CO_2_ sink, and the total *NEE* was −1085.1 g CO_2_·m^−2^. Liao et al. [39] reported that the carbon absorption capacities of *Spartina alterniflora*, *Phragmites australis*, and *Scirpus mariqueter* were 5096, 3410 and 770 g CO_2_·yr^−1^, respectively. Clearly, the CO_2_ absorption capacity of *Phragmites australis* is very high.

### Response of *R*_*eco*_ to temperature

The exponential function given in Eq. 2 is used to describe the relationship between *R*_*eco*_ and air temperature. Table 3 shows the relationship between nighttime *R*_*eco*_ and air temperature in the various ecosystems. Overall, nighttime *R*_*eco*_ increases exponentially with the air temperature. The relationship between *R*_*eco*_ and the air temperature varies across ecosystems and month-to-month (i.e., growth stages) in an ecosystem. Overall, the values of the coefficient of determination (R^2^) were not high in all of the ecosystems. This is caused by the uncertainties in the measured nighttime CO_2_ fluxes. The nighttime CO_2_ flux data are not very effective or reliable because of the low turbulence and stable atmospheric stratification [20, 21]. Compared to other ecosystems, the R^2^ values were lower in sandy desert, desert steppe, and Gobi ecosystems. There was little variations of the nighttime *R*_*eco*_ because commonly few vegetation living in these ecosystems and also because of the low moisture and organic content. In addition the night temperature changed rapidly in large gradient. All the above reasons are coupled to properly explain why the fitting of *R*_*eco*_ and temperature is not satisfactory occasionally. For the vegetable field, orchard and wetland ecosystems, the results fit best in September and most poorly in July. For the maize cropland, the best fit occurred during the filling stage and the poorest fit occurred during jointing. This shows that the effect of temperature on *R*_*eco*_ in the strong growth month or stage is higher than other months or stages.

**Table 3:** The relationship between nighttime ecosystem respiration and air temperature for different ecosystems.

sites	Month/Stage	Fitting equation	R^2^	Q_10_
Vegetable field	June	Reco = 0.0619exp(0.047*T)	0.119	2.05
	July	Reco = 0.113exp(0.0336*T)	0.083	1.40
	August	Reco = 0.0733exp(0.0548*T)	0.206	1.73
	September	Reco = 0.0697exp(0.0498*T)	0.265	1.65
Orchard	June	Reco = 0.0841exp(0.0571*T)	0.235	1.77
	July	Reco = 0.0987exp(0.0552*T)	0.210	1.74
	August	Reco = 0.1014exp(0.0451*T)	0.234	1.57
	September	Reco = 0.1287exp(0.0323*T)	0.375	1.38
Wetland	July	Reco = 0.0741exp(0.0402*T)	0.132	1.49
	August	Reco = 0.0605exp(0.0437*T)	0.175	1.55
	September	Reco = 0.048exp(0.0404*T)	0.305	1.50
Desert steppe	June-September	Reco = 0.0028exp(0.0235*T)	0.009	1.26
Gobi	June-September	Reco = 0.0026exp(0.043*T)	0.021	1.54
Sandy Desert	June-September	Reco = 0.0036exp(0.00572*T)	0.021	1.77
Maize cropland	seedling stage	Reco = 0.0316exp(0.0718*T)	0.312	2.05
	jointing stage	Reco = 0.1229exp(0.0392*T)	0.126	1.48
	filling stage	Reco = 0.1055exp(0.056*T)	0.512	1.75
	mature stage	Reco = 0.1052exp(0.0407*T)	0.293	1.50

The temperature sensitivities of the ecosystem (Q_10_) also have different values. Using Eq. 3, the Q_10_ values were estimated to be 1.26, 1.54, and 1.77 in the desert steppe, Gobi, and sandy desert ecosystems, respectively, during the observation period. The Q_10_ value ranged from 1.40 to 2.05 for the vegetable field, 1.38 to 1.77 for the orchard, 1.49 to 1.55 for the wetland and 1.48 to 2.05 for the maize cropland ecosystem. These values are similar to the Q_10_ value (1.5) based on air temperature at a global scale (excluding the wetlands) [40]. High Q_10_ values are observed in June for the vegetable field and orchard and in August for the wetland. Low Q_10_ values occurred in July for the vegetable field and wetland and in September for the orchard. For the maize cropland, high Q_10_ values were observed in the seedling stage, and low Q_10_ values in the jointing stage.

Thus, responses of *R*_*eco*_ to the environment vary depending on the ecosystems [41–43]. *R*_*eco*_ is regulated by numerous components, such as the respiration in the stem [44, 45], branch [46], root [47, 48] and foliage [46], microorganisms [49], decomposition of litter [50, 51], soil organic carbon [47, 48] and coarse wood debris [52]. Most researchers divide *R*_*eco*_ into respiration from the vegetation and from the soil [32, 53]. Given the low vegetation coverage in the desert, desert steppe and Gobi ecosystems, soil respiration dominated these ecosystems’ respiration. When vegetation coverage is relatively low in June (or the seedling stage), soil respiration plays an important role in *R*_*eco*_ and leads to high Q_10_ values. Low Q_10_ values occur in July and during the jointing stage when vegetation grows vigorously, which increases the proportion of *R*_*eco*_ derived from vegetation. In the orchard ecosystem, little change was observed in the apple trees during the observation period, and low Q_10_ values occurred in September with the apple harvest.

### Response of *NEE* to *PAR*

*PAR* is the most important factor for regulating daytime ecosystem CO_2_ exchanges during the growing season [53]. We used Eq. 4 to describe daytime *NEE* in response to *PAR*. The data were analyzed by month in the orchard, wetland, and vegetable field ecosystems and by growth stage in the maize cropland.

**Fig 4:**
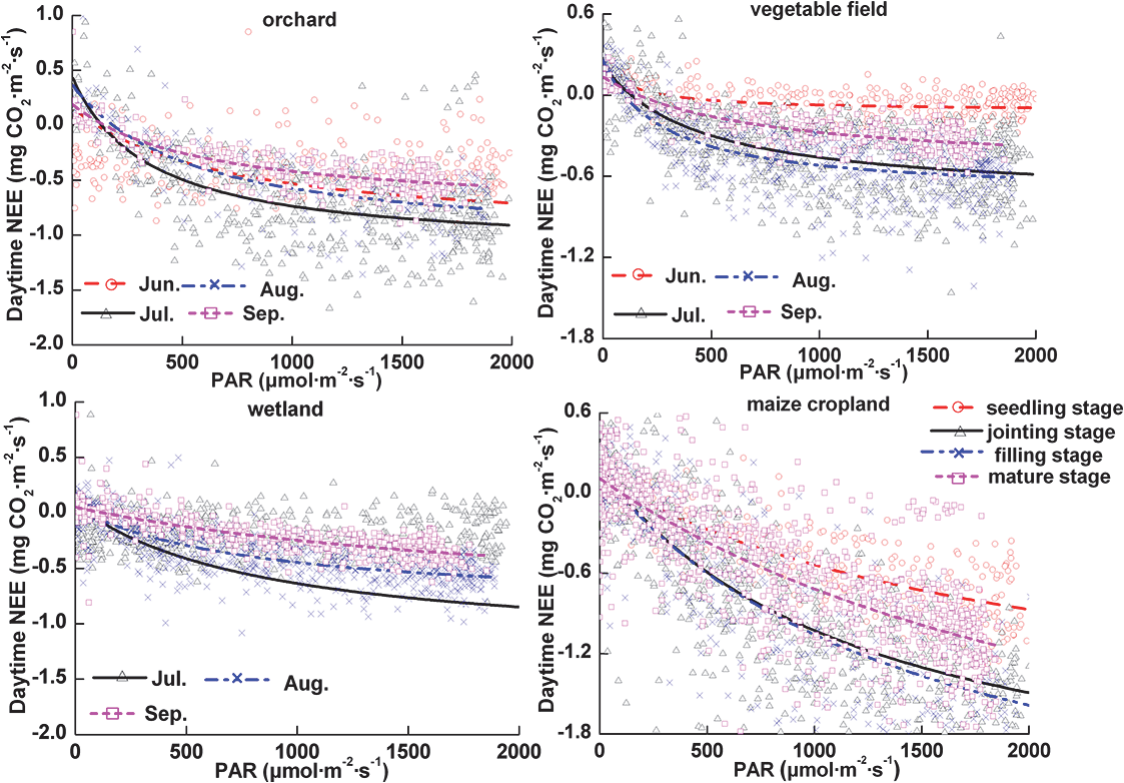
Response of *NEE* to *PAR* in different ecosystems.

Fig. 4 shows the relationship between daytime *NEE* and *PAR* in the vegetable field, orchard, wetland and maize cropland ecosystems. This figure shows that the CO_2_ flux gradually decreases with increases in *PAR*. Furthermore, the CO_2_ flux decreases linearly with low solar radiation (*PAR*<500 μmol·m^−2^·s^−1^) in all months or growth stages in all ecosystems; the rate of decrease gradually decreases until the CO_2_ flux stabilizes. The light saturation point varies with different vegetation. When *PAR* is greater than 1000 μmol·m^−2^·s^−1^, the CO_2_ flux became more stable in the vegetable field and wetland ecosystems. The *PAR* value was greater than 1600 μmol·m^−2^·s^−1^ and the carbon absorption rate remained stable in the orchard. However, as *PAR* values increased toward their maximum of 2000 μmol·m^−2^·s^−1^, carbon assimilation continued to increase in the maize cropland.

Table 4 shows the daily variation of the light response parameters α, *P*_*max*_ and *R*_*eco*_ in different ecosystems. In general, the seasonal patterns of these parameters closely followed the vegetation phenology. Additionally, there were some differences between the light response parameters of the different species. Previous studies found that *P*_*max*_ first increased and then decreased following the processes of crop growth, development and senescence [32]. We find similar results in our experiment. During the observation period, *P*_*max*_ shows seasonal trends in parallel with the vegetable growth. The value increased with the growth of leaves and reached a maximum in July; it then began to decrease as fruit ripening or foliage turned brown in the vegetable field, wetland, and orchard ecosystems. However, *P*_*max*_ continuously increased during the observation period in the maize cropland. This is because the maize was in the growth stage during the entire observation period. The *P*_*max*_ values ranged from 0.337 to 0.991 mg CO_2_·m^−2^·s^−1^ for the vegetable field; 0.977 to 1.584 mg CO_2_·m^−2^·s^−1^ for the orchard; 0.877 to 1.282 mg CO_2_·m^−2^·s^−1^ in the wetland; and 1.121 to 4.357 mg CO_2_·m^−2^·s^−1^ in the maize ecosystem (Table 4). These results clearly demonstrate that the peak *P*_*max*_ varies across ecosystems. The peak *P*_*max*_ was highest in maize cropland, followed in decreasing order by the orchard, wetland, and vegetable field ecosystems.

**Table 4:** Seasonal dynamics of light-response parameters for different ecosystems.

sites	Month/Stage	*P*_*max*_	*K*_m_	*R*_*eco*_	α	*R* ^*2*^
Vegetable field	June	0.337	156	0.219	0.0022	0.319
	July	0.991	450	0.223	0.0022	0.497
	August	0.975	402	0.188	0.0024	0.636
	September	0.685	675	0.134	0.0010	0.662
Orchard	June	1.171	694	0.160	0.0017	0.580
	July	1.580	348	0.439	0.0046	0.484
	August	1.456	540	0.369	0.0027	0.619
	September	0.977	604	0.189	0.0016	0.694
Wetland	July	1.282	418	0.062	0.0031	0.613
	August	0.877	403	0.038	0.0022	0.600
	September	0.935	1093	0.047	0.0009	0.644
Maize cropland	1	2.013	2055	0.124	0.0010	0.680
	2	2.725	1220	0.202	0.0022	0.708
	3	3.075	1509	0.168	0.0020	0.696
	4	3.153	2822	0.110	0.0011	0.671

1, 2, 3, and 4 represent the seedling stage, jointing stage, filling stage, and mature stage, respectively. The units of *P*_*max*,_ *K*_m_, *R*_*eco*_ and α are mg CO_2_·m^−2^·s^−1^, μmol·m^−2^·s^−1^, mg CO_2_·m^−2^·s^−1^, mg CO_2_· μmol^−1^, respectively.

The apparent quantum yield (α), calculated via Eq. 5, is a basic parameter of photosynthetic CO_2_ absorption, light utilization and material productivity. In general, the α value changes with the growth stage for all vegetation types, and a higher α value is presented during the months or stages in which plants grow rapidly. During the observation period, α first increased, reached a maximum in July or August for all vegetation types, and then decreased.

The α reported from our site is comparable to that found in other studies. The α value for the vegetable field ranged from approximately 0.0010 to 0.0024 mg CO_2_·μmol^−1^ and is similar to the value for dry croplands, which is between approximately 0.0009 and 0.0022 mg CO_2_·μmol^−1^ [54]. Flanagan et al. [55] reported α for a beech forest ranging from 0.0009 to 0.0013 mg CO_2_·μmol^−1^; this value is lower than that reported for the orchard in this study. Adequate water and fertilizer conditions may have led to the high α value. Zhao et al. [37] found the α value of an alpine wetland to be 0.0056 μmol·m^−2^·s^−1^ and 0.0082 μmol·m^−2^·s^−1^ in July and August, respectively. The value of the α of the wetland in this study is significantly higher than the value of the alpine wetland reported by Zhao et al., which could be due to the difference in hydrothermal conditions. The annual mean air temperature recorded at the alpine wetland (−1.7°C) is lower than in our study. Regarding the maize cropland ecosystem, the α value ranged from 0.0010 to 0.0022 mg CO_2_·m^−2^·s^−1^ and is similar to the value reported in previous studies. Suyker et al. [25] reported an α value of maize ranging from 0.02 to 0.04 μmol·m^−2^·s^−1^(0.0009–0.0018 mg CO_2_·m^−2^·s^−1^). Lei & Yang [53] reported a value of approximately 0.005 μmol·m^−2^·s^−1^ (0.0002 mg CO_2_·m^−2^·s^−1^) during the early growth stage and 0.065 μmol·m^−2^·s^−1^(0.0029 mg CO_2_·m^−2^·s^−1^) during the rapid growth stage for maize.

The variation in *R*_*eco*_ is similar to the variation in α. *R*_*eco*_ gradually increased and reached a maximum in July and then began to decrease in vegetable field, wetland, and orchard ecosystems in parallel with the temperature. For the maize cropland, the maximum *R*_*eco*_ was reached during the jointing stage. At this stage, the temperature is at its highest and maize is in its vigorous growth stage. The temperature is an important factor for *R*_*eco*_, and *R*_*eco*_ increases with temperature [53]. The pattern of *R*_*eco*_ is similar to the daytime *R*_*eco*_ derived from the light-response curve.

The R^2^ between *NEE* and *PAR* are significant in the main growth season of the vegetable field, orchard, wetland and maize cropland (Table 4). This means that the *PAR* and *NEE* fit well. The lowest R^2^ was 0.319 in June in the vegetable field. The transplanted vegetation were very small in June, and the site was nearly a bare field; also, the utilization of light was very low. These facts caused the poor fit in June in the vegetable field. In other ecosystems, the R^2^ values were high, especially in the maize cropland, which had a R^2^ greater than 0.670 for the entire growth stage.

## Conclusions

The CO_2_ flux in various ecosystems in the Zhangye oasis area were measured between June and September 2012 using the EC technique. The results demonstrate that:

1. Diurnal variations in the CO_2_ flux exhibit some differences across ecosystems. Gobi, sandy desert, and desert steppe ecosystems have smaller amplitude CO_2_ flux values compared to the other ecosystems due to their lower vegetation coverage. The values of *NEE* are close to zero; however, CO_2_ absorption is observed in the daytime. The diurnal variations in the CO_2_ flux are obvious in the wetland, vegetable field, orchard, and maize cropland ecosystems. Due to the differences in carbon absorption capacities, the amplitudes of carbon are significantly different among the different vegetation types.
2. The daily distributions of *NEE*, *GPP* and *R*_*eco*_ of Gobi, sandy desert and desert steppe ecosystems are similar, and there are significant daily variations in the vegetable field, orchard, wetland, and maize cropland ecosystems, that are closely related to the vegetation development and phenology. The values of *NEE*, *GPP*, and *R*_*eco*_ fluctuated around zero in the Gobi, sandy desert and desert steppe. Additionally, during the observation period, *GPP*, *NEE*, and *R*_*eco*_ have only one peak period in the vegetable field, orchard, and wetland and maize cropland ecosystems. In short, all of the ecosystems acted as a carbon sink during the observation period. The integrated carbon absorption capacity is as follows: maize cropland > wetland > orchard > vegetable field> sandy desert> Gobi> desert steppe.
3. Using exponential curve fitting, we demonstrated that nighttime respiration increases exponentially with temperature in some ecosystems, and the results varied both across ecosystems and month-to-month within a given ecosystem. In addition, high Q_10_ values appeared in June or seedling stage when vegetation coverage was relatively low, and low Q_10_ values occurred in July or jointing stage when plants grew vigorously.
4. *PAR* is the most important factor for regulating daytime ecosystem CO_2_ exchange in the vegetable field, orchard, wetland, and maize cropland ecosystems. The responses of different ecosystems to *PAR* are not the same. Overall, *PAR* is the dominant factor for controlling photosynthesis under low solar radiation, and the CO_2_ assimilation rate increases slowly in the presence of high solar radiation. The light saturation point was approximately 1000 μmol·m^−2^·s^−1^ for the vegetable field and wetland ecosystems, 1600 μmol·m^−2^·s^−1^ for the orchard, and more than 2000 μmol·m^−2^·s^−1^ for the maize cropland. Seasonal variations in the light response parameters, α, *P*_*max*_ and *R*_*eco*_, in different ecosystems closely follow vegetation phenology. In general, the values changed with the growth stage for all of the vegetation types, and higher values occurred during months or stages with rapid growth.

## Supporting Information

S1 Data All the metadata in our study. (XLSX)
