# Correction: Diurnal and Seasonal Variations in Carbon Dioxide Exchange in Ecosystems in the Zhangye Oasis Area, Northwest China

**DOI:** 10.1371/journal.pone.0130243

**Published:** 2015-06-04

**Authors:** Lei Zhang, Rui Sun, Ziwei Xu, Chen Qiao, Guoqing Jiang

Table 2, “Total *GPP*, *R*_*eco*_, and *NEE* for different ecosystems during the observation period (June 10—September 14, and, in wetland, June 26—September 14)”, incorrectly appears as identical to Table 3. Please see the corrected Table 2 here.

**Table 2:** Total *GPP*, *R*_*eco*_, and *NEE* for different ecosystems during the observation period (June 10—September 14, and, in wetland, June 26—September 14)

	Orchard	Vegetable field	Gobi	Sandy desert	Desert steppe	Wetland	Maize cropland
NEE (g CO_2_·m^2^)	-1050.3	-468.1	-67.9	-129.6	-17.8	-1085.1	-2342.6
R_eco_ (g CO_2_·m^2^)	2362.5	1839.6	70.4	147.9	194.4	1139.7	2031.4
GPP (g CO_2_·m^2^)	3412.8	2307.7	138.3	277.5	212.2	2224.8	4374.0
GPP/R_eco_	1.44	1.25	1.96	1.88	1.09	1.95	2.15
